# Assessment of the value of adjuvant radiotherapy for treatment of gastric adenocarcinoma based on pattern of post-surgical progression

**DOI:** 10.1186/s12957-021-02304-4

**Published:** 2021-07-08

**Authors:** Peng Wang, Haihua Zhou, Gaohua Han, Qingtao Ni, Shengbin Dai, Junxing Huang, Chunlei Dai, Lei Yu

**Affiliations:** 1 Department of Oncology, Hospital Affiliated 5 to Nantong University (Taizhou People’s Hospital), 399 Hailing South Road, Taizhou, Jiangsu China; 2 Department of General Surgery, Hospital Affiliated 5 to Nantong University (Taizhou People’s Hospital), Taizhou, 225300 Jiangsu China; 3 Department of Nuclear Medicine, Hospital Affiliated 5 to Nantong University (Taizhou People’s Hospital), Taizhou, 225300 Jiangsu China

**Keywords:** Gastric carcinoma, Radical resection (R0), Local recurrence, Adjuvant radiotherapy

## Abstract

**Purpose:**

To assess the value of adjuvant radiotherapy for treatment of gastric adenocarcinoma and to investigate subgroups of patients suitable for adjuvant radiotherapy.

**Methods and materials:**

Data from 785 patients with gastric adenocarcinoma who had undergone D1/D2 radical resection and adjuvant chemotherapy were collected, the site of first progression was determined, and the relationship between the rate of local recurrence and clinicopathologic features was analyzed.

**Results:**

By the end of the follow-up period, progression was observed in 405 patients. Local recurrence was observed as the first progression in 161 cases. The local recurrence rate was significantly lower than the non-local progression rate (20.5% vs 31.5%, p=0.007). Multivariate Cox regression analysis showed a significant relationship among degree of differentiation, T stage, N stage, and rate of local recurrence.

**Conclusions:**

Not all patients with gastric carcinoma required adjuvant radiotherapy. However, patients with poorly differentiated cancer cells, advanced T stage (T3/T4), and positive lymph nodes, which included patients in the T4N1-2M0 subgroup, were recommended for adjuvant radiotherapy.

## Introduction

Gastric cancer is prevalent, where it is responsible for the third-most cancer-related deaths. Treatment of gastric cancer consists of a comprehensive treatment model based on surgery. However, there is no standard model for postoperative adjuvant therapy for gastric cancer, and postoperative treatment plans are comprised primarily of chemotherapeutic approaches. The SWOG 9008/INT-0116 studies showed that the survival rate and local control rate of gastric cancer were significantly improved following gastric cancer surgery (IB-IV(M0)), and there have been many advances in postoperative adjuvant therapy following gastric cancer surgery [1]. Use of adjuvant chemoradiotherapy following gastric cancer surgery has increased significantly in the USA and Canada [2, 3]. Postoperative radiotherapy and chemotherapy have been established as the postoperative treatment standards in the USA for patients with gastric cancer. However, this treatment mode is only recognized internationally as suitable for patients with gastric cancer undergoing D0 radical surgery [4]. The benefits of adjuvant radiotherapy following D1/D2 radical surgery for treatment of gastric cancer have not been characterized.

Radiotherapy is a local treatment method that can kill and damage tumor cells in the radiation field. Radiotherapy can only be used if the recurrent lesion is within the radiation field. According to the ICRU50 report definition, postoperative adjuvant radiotherapy for gastric cancer should target the tumor bed, the stomach area, anastomotic regions, regional lymphatic drainage areas, and the celiac axis (including perigastric, peritoneal, hepatic, gastroduodenal, splenic, and peripancreatic lymph nodes) [5]. Therefore, the postoperative recurrence location of gastric cancer plays a critical role in selection of postoperative adjuvant therapy. In this study, 785 patients with gastric cancer treated in our hospital from January 2005 to January 2015 who had received D1 or D2 radical surgery and had follow-up data were analyzed retrospectively. We evaluated postoperative adjuvant radiotherapy for treatment of gastric cancer by observing whether the site of first progression was within the appropriate target area. Furthermore, clinicopathological factors were analyzed to determine risk factors for postoperative local recurrence of gastric cancer and to identify indications for use of postoperative adjuvant radiotherapy.

## Material and methods

### Inclusion and exclusion criteria

Patients with gastric adenocarcinoma (including cardiac adenocarcinoma) who underwent radical surgical resection (R0) in our hospital from January 2005 to January 2015 were enrolled. Authors had access to information that could not identify individual participants during or after data collection. Clinical information about gastric adenocarcinoma cases was extracted from the hospital information system (HIS) and medical records at the tertiary hospital. Information from medical records was compiled in abstracted evaluations. All evaluations were reviewed and scored by two clinical investigators who developed and employed a coding guide based on the International Statistical Classification of Diseases and Related Health Problems (ICD) criteria to determine if the gastric adenocarcinoma labeling was consistent with the standard international diagnostic criteria of gastric adenocarcinoma. Postoperative adjuvant chemotherapy, immunotherapy, and other comprehensive treatments were performed. Patients who underwent palliative surgery (R1 or R2 resection), D0 radical surgery, neoadjuvant radiotherapy, postoperative adjuvant radiotherapy, or had experienced gastric cancer recurrence and subsequent treatment were excluded from this study.

### Follow-up

Regular follow-up was carried out in accordance with the institutional surveillance strategy, including medical history, physical examination, tumor biomarkers, serum biochemical, CT scans of the chest, abdomen and pelvis (or positron-emission tomographic (PET) scans if necessary and within a budget limit) at each visit, as well as endoscopy. Patients were followed up every 3 months for the first 2 years, every 6 months until 5 years, and yearly thereafter.

### Confirmation of initial site of progression

Patients who had experienced recurrence or metastasis were located through a systematic review of medical records of inpatients and outpatients. Patient imaging data in our PACS (Picture Archiving and Communication Systems) and external information were reviewed, including digestive tract images, chest and abdomen CT scans, brain and spine MRI scans, and whole-body bone scans. The imaging department and oncologists read the films and recorded and analyzed the first site of progression. Some cases also had biopsy pathology results if necessary. Only first recurrences or metastases were included in the statistical analysis.

### Classification of first progression sites

Postoperative progression of gastric cancer was categorized as regional recurrence, intraperitoneal metastasis, or extraperitoneal distant metastasis. Regional recurrence included residual gastric cancer, anastomotic recurrence, recurrence of the original tumor, duodenal stump recurrence, and lymph node metastasis at stations [1]. Intraperitoneal metastases included peritoneal implantation (carcinogenic ascites, mesenteric carcinoma nodules) and intra-abdominal viscera metastasis (such as liver, spleen, adrenal gland, and ovary). Extraperitoneal metastases included lung, brain, bone, bone marrow, and extraperitoneal lymph nodes. The first progression was local recurrence, which was the target area of postoperative adjuvant radiotherapy. Intraperitoneal and extraperitoneal metastases were considered non-local progression, and were not candidates for local adjuvant radiotherapy.

### Statistical methods

The relationships between clinical parameters and overall recurrence/local recurrence were analyzed using the χ^2^ test, and independent factors that influenced local recurrence were determined using Cox multivariate regression analysis. P<0.05 was considered statistically significant, and all analyses were performed using SPSS 19.0 software package.

## Results

### Patient characteristics

A total of 1342 gastric cancer surgeries were performed in our hospital over 10 years, and 132 cases of gastric cancer were treated with adjuvant chemotherapy in our hospital following surgery in another hospital (with detailed pathological reports). A total of 785 patients had complete follow-up data as of December 2018, with a median follow-up time of 83 months. Among the 785 patients with gastric cancer, 496 were males and 289 were females (1.7:1 ratio). The minimum age was 19 years, the maximum age was 76 years, and the median age was 54 years. This study included 256 cases of cardiac adenocarcinoma (gastric esophageal junction cancer), 166 cases of gastric body cancer, and 363 cases of gastric sinus cancer.

Five hundred forty-eight patients underwent subtotal gastrectomy (including 305 cases of proximal subtotal gastrectomy and 243 cases of distal subtotal gastrectomy), and 237 underwent total gastrectomy, of whom 65 underwent other resection (all of which were T4). Four hundred fifty-four cases were treated with D1+ radical resection, and 331 cases were treated with D2 radical resection. Most patients with stage IB cancer did not receive chemotherapy following surgery. A small number of patients with stage IB cancer with high-risk factors (well cancer cell differentiation, vascular cells, high Ki-67 expression) and patients with stage II or more advanced cancer received postoperative adjuvant chemotherapy. The chemotherapy administered was 5-FU + OXA. Patients with stage III/IV cancer were administered taxanes via peritoneal perfusion as the primary adjuvant chemotherapy. Although peritoneal perfusion chemotherapy was recommended by Consensus of Chinese expert, and the result from PHOENIC-GC has also indicated that the patients could benefit from peritoneal perfusion chemotherapy [1]. None of the patients in this group received postoperative adjuvant radiotherapy or concurrent radiotherapy.

### Postoperative pathology

Patient tumors included 752 cases of adenocarcinoma (121 cases of well-differentiated papillary or tubular adenocarcinoma, 154 cases of moderately differentiated mucinous adenocarcinoma, and 477 cases of poorly differentiated carcinoma, of which 120 cases were signet ring cell carcinoma), 20 cases of acanthoadenocarcinoma, and 13 cases of adenocarcinoma associated with neuroendocrine carcinoma. The tumor had invaded the intrinsic layer and submucosal layer (T1) in 25 cases. Invasion of the muscle layer, but not the serosal layer (T2), occurred in 305 cases, and 455 tumors had broken through the serosal layer or exhibited peripheral viscera invasion (T3, T4). The number of lymph nodes affected ranged from 5 to 46, and 713 cases exhibited lymph node metastasis. Of these 713 cases, 638 patients had less than 7 positive lymph nodes (N1, N2) and 75 patients had more than 7 positive lymph nodes (N3). Sixty-nine cases were stage IB, 228 cases were stage II, 393 cases were stage III, and 95 cases were stage IV, which indicated that middle-to-late stages accounted for the majority of cases.

### Progression rate and initial progression location

By the date of follow-up (December 2018), 405 patients had experienced recurrence or metastasis, with a progression rate of 51.6%, and the median time to first progression was 37 months. These included 315 cases of single site recurrence or metastasis and 90 cases of simultaneous progression of 2 or more sites. There were 161 cases of regional recurrence and 244 cases of non-local recurrence or metastasis (96 cases of intraperitoneal metastasis and 148 cases of extraperitoneal distant metastasis). Local recurrence manifested primarily as regional lymph node metastasis, followed by recurrence of anastomosis or/and residual stomach cancer. A small number of patients experienced tumor bed recurrence, and duodenal stump recurrence was rare. Intra-abdominal viscera metastasis was mostly observed in the liver, ovary, and spleen. Extraperitoneal distal metastasis was most common in the lungs, mediastinum, neck, and supraclavicular lymph nodes. Bone was the third most common metastatic site (shown in Table 1). The local recurrence rate was significantly lower than the non-local progression rate (intraperitoneal metastasis+ distant metastasis) (20.5% vs 31.5%, p=0.007).

**Table 1:** Detailed information of the first progression pattern after gastric cancer surgery

The location of the first progress	Number	The proportion of all cases(%)	The proportion of advanced cases(%)
Local recurrence	161		
Anastomotic recurrence	53	6.8	13.8
Regional lymph node metastasis	67	8.5	17.4
The residual stomach cancer	23	2.9	5.9
Duodenal stump recurrence	13	1.6	3.4
The abdominal wall incision recurred	5	0.6	1.3
Peritoneal dissemination	37		
Cancerous ascites	30	3.8	7.8
Mesenteric tubercle	7	0.9	1.8
Abdominal visceral metastasis	59		
Liver	42	5.4	10.9
Ovary	12	1.5	3.1
Adrenal gland	5	0.6	1.3
Distant metastasis	148		
Pulmonary ± mediastinal lymph nodes	52	6.6	13.5
Cervical and supraclavicular lymph nodes	37	4.7	9.6
Brain	23	2.9	6
Bone	39	5	10.1
Bone marrow	1	0.1	0.3

### Association of first site of progression with clinicopathological parameters

The results of subgroup analysis based on clinicopathological parameters are shown in Table 2. The overall progression rate of cardiac carcinoma was similar to that of gastric body/sinus carcinoma. There was no statistically significant difference in local recurrence rate (p=0.54) or non-local recurrence rate (p=0.11) between cardiac carcinoma and gastric body/sinus carcinoma. The overall progression rate of patients with low differentiation was significantly higher than that of patients with moderate-to-well differentiation. The local recurrence rate in patients with low differentiation was significantly higher than that in patients with moderately-to-well differentiated cancer (p=0.001). In contrast, the non-local metastasis rate did not differ with level of differentiation (p=0.39). The total progression rate of patients with stage III/IV cancer was significantly higher than that of patients with stage I/II cancer. The local recurrence rate of patients with stage III/IV cancer was significantly higher than that in patients with stage I/II cancer (p=0.03), and the nonlocal transfer rate was significantly higher in patients with stage III/IV cancer than that in patients with stage I/II cancer (p=0.004).

**Table 2:** Probability of first failure sites under different clinicopathological parameters (%)

First failure site	Cancer of the stomach area (n, %)		The degree of differentiation of cancer cells (n, %)		TNM staging (n, %)	
	Cardiac cancer	Gastric body/gastric sinus	Moderate-well differentiation	Low differentiation	I/II	III/IV
Local recurrence	71 (27.7)	90 (17.0)	41 (14.9)	120 (25.1)	49 (16.5)	112 (22.9)
Non-local recurrence	123 (47.4)	121 (22.8)	87 (29.3)	157 (32.2)	73 (24.6)	171 (35)

### Single factor analysis of local recurrence following gastric cancer surgery

Univariate analysis of clinical or pathological factors that may have influenced postoperative local recurrence of gastric cancer was performed (Table 3). The results showed that gender, age, gastric cancer site, surgical resection scope, and presence or absence of endoscopic vascularized tumor thrombus were not associated with local recurrence rate (p>0.05). In contrast, degree of differentiation, T stage, and TNM clinical stage of cancer cells were significantly correlated with postoperative local recurrence rate of gastric cancer (p<0.05). In addition, while patients with stage N2 and N3 cancer had an increased local recurrence rate, the rate of distant metastasis was higher.

**Table 3:** Single factor analysis of local recurrence after gastric cancer surgery

Factors	Number of cases (%)	Local recurrence rate	*P*
Gender			0.49
Male	98/496	19.7	
Female	63/289	21.8	
Age			0.740
<55	41/208	19.7	
>55	120/577	20.8	
Cancer of the stomach area			0.721
Cardiac cancer	71/356	19.9	
Gastric body/gastric sinus cancer	90/429	20.9	
Histological classification			0.001
Well differentiation	12/121	9.9	
Moderate differentiation	29/154	18.8	
Low differentiation (including signet ring cell carcinoma)	120/477	25.2	
Surgical resection range			0.487
Partial gastrectomy	116/548	21.2	
Total gastric resection	45/237	19	
Vascular tumor emboli			0.577
With	59/273	21.6	
Without	102/512	19.9	
T Staging			≤0.001
T1	1/25	4	
T2	44/305	14.4	
T3	82/390	21	
T4	34/65	52.3	
N Staging			0.013
N0	11/72	15.3	
N1	64/382	16.8	
N2	65/256	25.4	
N3	21/75	28	
TNM Clinical staging			0.010
Ib	17/69	24.6	
II	32/228	14	
III	84/393	21.4	
IV(M0)	28/95	29.5	

### Single factor analysis of local recurrence following cardiac cancer surgery

Univariate analysis of clinical or pathological factors that may have influenced postoperative local recurrence of cardiac cancer was performed (Table 4). Among the 256 cardiac cancer patients, 146 were males and 110 were females (1.3:1 ratio). The tumors included 256 cases of adenocarcinoma (6 cases of well differentiation; 11 cases of moderate differentiation; and 156 cases of low differentiation). The tumor had invaded the intrinsic layer and submucosal layer (T1) in 7 cases. Invasion of the muscle layer, but not the serosal layer (T2), occurred in 102 cases, and 147 tumors had broken through the serosal layer or exhibited peripheral viscera invasion (T3, T4). The results showed that gender, age, and presence or absence of endoscopic vascularized tumor thrombus were not associated with local recurrence rate (p > 0.05). In contrast, degree of differentiation, T stage, and TNM clinical stage of cancer cells were significantly correlated with postoperative local recurrence rate of cardiac cancer (p < 0.05). The local recurrence rate in patients with low differentiation was significantly higher than that in patients with moderately-to-well differentiated cancer (p=0.005), in addition, while patients with stage N2 and N3 cancer had an increased local recurrence rate.

**Table 4:** Single factor analysis of local recurrence after cardiac cancer surgery

Factors	Number of cases (%)	Local recurrence rate	*P*
Gender			0.671
Male	42/146	28.8	
Female	29/110	26.4	
Age			0.939
<55	20/73	27.4	
>55	51/183	27.9	
Histological classification			0.005
Well differentiation	6/49	12.2	
Moderate differentiation	11/51	21.6	
Low differentiation (including signet ring cell carcinoma)	54/156	34.6	
Vascular tumor emboli			0.641
With	22/85	25.9	
Without	49/171	28.7	
T staging			<0.001
T1	1/7	14.3	
T2	16/102	15.7	
T3	31/112	27.7	
T4	23/35	65.9	
N staging			0.022
N0	3/22	13.6	
N1	30/128	23.4	
N2	27/84	32.1	
N3	11/22	50.0	
TNM clinical staging			0.014
Ib	3/21	14.3	
II	16/78	20.5	
III	39/131	29.8	
IV(M0)	13/26	50.0	

### Multivariate Cox regression analysis of postoperative local recurrence of gastric cancer and cardiac cancer

Multivariate Cox regression analysis showed that poorly differentiated cancer cells and more advanced T staging (T3, T4) were associated with a significantly higher local recurrence rate. There were no statistically significant differences in lymph node metastasis. However, subgroup analysis showed that the local recurrence rate of patients with stage N2 cancer was high, and patients with stage N3 cancer experienced more distant metastasis than local recurrence (Tables 5 and 6). In the multivariate analysis, TNM stage did not show any correlation with local recurrence rate.

**Table 5:** Multivariate analysis of local recurrence after gastric cancer surgery

Factors	Number of cases (%)	HR (95% CI)	*P*
Histological classification			
Moderate-to-well differentiation (reference)	41	1.00	
Low differentiation (including signet ring cell carcinoma)	120	2.15 (1.25–3.86)	0.007
T staging			0.001
T1+T2 (reference)	45	1.00	
T3	82	3.99 (1.77–9.02)	0.001
T4	34	3.51 (0.64–8.94)	≤0.001
N staging			0.24
N0 (reference)	11	1.00	
N1	64	3.28 (1.02–10.57)	0.06
N2	65	3.16 (1.01–9.88)	0.04
N3	21	3.31 (0.69–15.83)	0.33

**Table 6:** Multivariate analysis of local recurrence after cardiac cancer surgery

Factors	Number of cases (%)	HR (95% CI)	*P*
Histological classification			
Moderate-to-well differentiation (reference)	17	1.00	
Low differentiation (including signet ring cell carcinoma)	54	2.02 (1.18–3.46)	0.010
T staging			0.001
T1+T2 (reference)	17	1.00	
T3	31	3.76 (1.81–7.81)	<0.001
T4	23	3.43 (1.24–9.49)	0.018
N staging			0.068
N0(reference)	3	1.00	
N1	30	2.79 (1.08–7.21)	0.034
N2	27	3.35 (1.12–10.02)	0.031
N3	11	3.05 (0.59–15.77)	0.183

## Discussion

Current treatment for gastric cancer typically involves surgery and laparoscopic gastrectomy [6, 7]. More than 60% gastric cancer patients experienced recurrence after surgery [8]. Postoperative local recurrence, regional recurrence, and distant metastasis are common following surgical intervention for gastric cancer. Peritoneal involvement and pre-treatment platelet-lymphocyte ratio were associated with poor overall survival in gastric cancer patients [9, 10]. Comprehensive measures were advised according to the pathophysiology of gastric cancer [11]. Approval of postoperative adjuvant radiotherapy for gastric cancer in the USA was based on two factors. First, adjuvant chemotherapy failed to reduce the local recurrence rate and improve survival in patients who had undergone surgical treatment for gastric cancer [12]. Second, the results of two clinical trials, including the INT 0116 study, showed that the median survival period of patients who underwent adjuvant chemoradiotherapy following gastric cancer surgery was significantly longer than that of patients who only underwent surgery [1, 13]. Therefore, postoperative chemoradiotherapy for gastric cancer has become the standard of postoperative treatment in North America. However, the INT 0116 study suffered from some limitations that prevented widespread recognition of the results regarding adjuvant chemoradiotherapy. The patients enrolled in the study ranged from stage IB to stage IV (M0). In addition, most of the patients in the study underwent D0 radical surgery, which accounted for 54% of the subjects in the study. Very few (10%) of the subjects underwent D2 surgery. The results showed that patients who underwent D0 surgical resection benefited from adjuvant chemoradiotherapy. Gastric cancer patients with D1 or D1 plus lymphadenectomy can benefit from postoperative radiotherapy [14]. However, D2 surgery has become a first line therapeutic approach in Asia and Europe [15–17]. Furthermore, the NCCN guidelines recommended D2 radical resection whenever possible. Therefore, the INT 0116 study did not reflect current guidelines for treatment of gastric cancer. In addition, the INT 0116 study used CF/5-FU as the chemotherapeutic strategy. This treatment approach was inefficient and was not representative of better chemotherapeutic drugs currently used to treat gastric cancer. Many studies have reported that adjuvant chemotherapy significantly reduced recurrence and prolonged survival [18, 19]. Furthermore, the INT 0116 study compared postoperative radiotherapy and chemotherapy to surgery alone. This resulted in an inability to determine whether curative effects were due to radiotherapy or chemotherapy. Previous reports showed that postoperative radiation alone did not prolong survival [20], so the survival benefit of chemoradiotherapy may derive from the addition of chemotherapy. Postoperative treatment of gastric cancer in Asian countries showed results that differed from the INT 0116 study. Standard D2 surgery and postoperative chemotherapy improved patient survival [21]. The 5-year survival rate of patients with locally advanced gastric cancer treated with surgery alone in Japan and South Korea reached 70%, which was far higher than the 42% 5-year survival rate report in the USA. Therefore, improvements in surgical methods and development of more effective chemotherapeutic drugs has resulted in better treatment of gastric cancer, and the postoperative local recurrence rate has been significantly reduced. Batista suggested that these improvements highlighted the need to evaluate whether postoperative adjuvant radiotherapy would also benefit patients undergoing D2 radical surgery [22].

A number of clinical trials have been designed to study the effects of adjuvant chemoradiotherapy following D2 radical resection of gastric cancer. A prospective Korean study (ARTIST) showed that patients with lymph node metastasis who received postoperative adjuvant chemoradiotherapy following D2 radical surgery exhibited longer disease-free survival time than those who received chemotherapy alone [23]. Kim et al. showed that chemoradiotherapy improved survival of patients with advanced stage gastric cancer (particularly stage III) to a greater extent than those who received chemotherapy alone [24]. Endostar combined with radiotherapy can inhibit tumor growth [25]. These studies suggested that postoperative radiotherapy may benefit patients with gastric cancer who are at high risk of recurrence. At present, D2 radical gastrectomy for treatment of gastric cancer is widely practiced. However, due to the high proportion of locally advanced gastric cancer, inclusion of postoperative adjuvant therapy is critical. The necessity of inclusion of adjuvant radiotherapy for treatment of Chinese patients with gastric cancer has not been determined. Furthermore, if it is not necessary for all patients, and the subgroups that need adjuvant radiation require further definition. Zhu et al. showed that postoperative adjuvant chemoradiotherapy prolonged disease-free survival to a greater extent than postoperative chemotherapy alone in patients with gastric cancer who underwent D2 radical surgery, but there was no difference in overall survival [26]. A recent meta-analysis showed that adjuvant chemoradiotherapy improved disease-free survival, to a greater extent than adjuvant chemotherapy in patients who underwent surgery to treat locally advanced gastric cancer. These findings indicated that the utility of postoperative adjuvant radiotherapy is still unclear [4, 27].

The degree of local recurrence following gastric cancer surgery determines the necessity for postoperative radiotherapy. Some studies have suggested that local recurrence occurred more frequently than peritoneal implantation metastasis and distant metastasis following surgery to treat gastric cancer [28–32]. Our study of 785 patients who received postoperative adjuvant chemotherapy resulted in 161 cases of local recurrence as the first progression and 244 cases of non-local recurrence as the first progression. This finding indicated that the local recurrence rate of patients who underwent D1/D2 radical surgery was lower than that of non-local recurrence, which was similar to the results of another study [13]. The reason for this result following D1/D2 radical surgery may have been because these procedures were thorough and standardized and resulted in adequate local removal. In contrast, drug selection may have resulted in the discrepancy between local and distant recurrence. These patients did not receive survival benefits from local radiotherapy. Therefore, only 140 cases (17.8%) were suitable for adjunctive radiotherapy, which was far lower than the 244 cases with non-local recurrence (31.1%). These results suggested that the progression pattern of patients with gastric cancer who underwent D1 and D2 radical surgery and postoperative standardized chemotherapy was mainly distant metastasis. A previous study showed that the postoperative progression pattern for early stage patients with gastric cancer was also mainly distant metastasis [33]. Not all patients with gastric cancer needed adjuvant radiotherapy following surgery. Multivariate analysis showed that patients with cancer cells with low differentiation, deep invasion (T3, T4), and lymph node metastasis had a high local recurrence rate, which was similar to results from previous studies [34, 35]. Furthermore, patients with stage N3 cancer were more prone to distant metastasis, but were not at increased risk for local recurrence. We concluded that the T2-3N3M0 and T4N2-3M0 subgroups among patients with stage IV cancer were at higher risk of distant metastases and were candidates for systemic chemotherapy. However, the local recurrence rate in the T4N1-2M0 subgroup was higher, which indicated that patients in this subgroup were candidates for postoperative adjuvant radiotherapy. There was no significant difference in the total survival between patients who received chemoradiotherapy and those who received adjuvant chemotherapy, but the side effects associated with chemoradiotherapy were significantly more severe than those associated with chemotherapy alone [1, 36]. Seventeen percent of the patients in the INT 0116 trial withdrew from the trial because of excessive radiation side effects. Postoperative adjuvant radiotherapy for treatment of gastric cancer should be used with caution.

However, there are still some limitations in this case search. For example, we found that most of the patients had completed chemotherapy, but due to the limitation of time and manpower, we failed to make further statistics on the completion rate of chemotherapy. Of course, in clinical practice, we strictly followed the requirements of the guidelines, reduced the amount of patients with side effects, and also observed that most of the patients had completed postoperative chemotherapy. Besides, it is also hard to provide the subgroup analysis in 65 patients of gastrectomy combined with other visceral resections since the surgical record has a long history. Because of the time span of patients’ inclusion and the different clinical stages according to different standards, it is inevitable that some bias may be caused in clinical practice and the results of this study. Though there are some limitations in this study, it is still expected that there are some interesting findings of this research. The local recurrence rate in patients with gastric cancer who received adjuvant chemotherapy (including systemic chemotherapy and peritoneal perfusion chemotherapy) following D1/D2 radical surgery was low, but the non-local recurrence/metastasis rate was higher. Therefore, most patients with gastric cancer did not need adjuvant radiotherapy.

## Conclusions

Our data suggested that patients with risk factors such as poor differentiation of cancer cells, advanced T staging, and positive lymph nodes were candidates for adjuvant radiotherapy. Patients with stage N3 gastric cancer were more likely to develop distant metastases, and the T4N1-2M0 subgroup was more likely to benefit from local radiotherapy.

## Data Availability

All relevant data are within the manuscript and its supporting information files.

## References

[1] Macdonald JS, Smalley SR, Benedetti J, Hundahl SA, Estes NC, Stemmermann GN, Haller DG, Ajani JA, Gunderson LL, Jessup JM, Martenson JA (2001). Chemoradiotherapy after surgery compared with surgery alone for adenocarcinoma of the stomach or gastro- esophageal junction. N Engl J Med.

[2] Kozak KR, Moody JS (2008). The survival impact of the intergroup 0116 trial on patients with gastric cancer. Int J Radiat Oncol Biol Phys.

[3] Coburn NG, Guller U, Baxter NN, Kiss A, Ringash J, Swallow CJ (2008). Adjuvant therapy for resected gastric cancer–rapid,yet incomplete adoption following results of intergroup 0116 trial. Int J Radiat Oncol Biol Phys.

[4] Min C, Bangalore S, Jhawar S (2014). Chemoradiation therapy versus chemotherapy alone for gastric cancer after R0 surgical resection: a meta-analysis of randomized trials. Oncology.

[5] Liu GF, Bair RJ, Bair E (2014). Outcomes for gastric cancer following adjuvant chemoradiation utilizing intensity modulated versus three-dimensional conformal radiotherapy. PLoS One.

[6] Choi H, Yang SY, Cho HS, Kim W, Park EC, Han KT (2017). Mortality differences by surgical volume among patients with stomach cancer: a threshold for a favorable volume-outcome relationship. World J Surg Onc.

[7] Wang Y, Wang Y, Wu W, Lu X, An T, Jiang J (2021). Laparoscopic gastrectomy plus D2 lymphadenectomy is as effective as open surgery in terms of long-term survival: a single-institution study on gastric cancer. World J Surg Onc.

[8] Liu D, Lu M, Li J, Yang Z, Feng Q, Zhou M, Zhang Z, Shen L (2016). The patterns and timing of recurrence after curative resection for gastric cancer in China. World J Surg Onc.

[9] Hultman B, Gunnarsson U, Nygren P, Sundbom M, Glimelius B, Mahteme H (2017). Prognostic factors in patients with loco-regionally advanced gastric cancer. World J Surg Onc.

[10] Zhang X, Zhao W, Yu Y, Qi X, Song L, Zhang C, Li G, Yang L (2020). Clinicopathological and prognostic significance of platelet-lymphocyte ratio (PLR) in gastric cancer: an updated meta-analysis. World J Surg Onc.

[11] Beeharry MK, Zhang TQ, Liu WT, Gang ZZ (2020). Optimization of perioperative approaches for advanced and late stages of gastric cancer: clinical proposal based on literature evidence, personal experience, and ongoing trials and research. World J Surg Onc.

[12] Hermans J, Bonenkamp JJ, Boon MC, Bunt AM, Ohyama S, Sasako M, van de Velde CJ (1993). Adjuvant therapy after curative resection for gastric cancer: meta-analysis of randomized trials. J Clin Oncol.

[13] Kim S, Lim DH, Lee J, Kang WK, MacDonald JS, Park CH, Park SH, Lee SH, Kim K, Park JO, Kim WS, Jung CW, Park YS, Im YH, Sohn TS, Noh JH, Heo JS, Kim YI, Park CK, Park K (2005). An observational study suggesting clinical benefit for adjuvant postoperative chemoradiation in a population of over 500 cases after gastric resection with D2 nodal dissection for adenocarcinoma of the stomach. Int J Radiat Oncol Biol Phys.

[14] Zhang N, Fei Q, Gu J, Yin L, He X (2018). Progress of preoperative and postoperative radiotherapy in gastric cancer. World J Surg Onc.

[15] Hartgrink HH, van de Velde CJ, Putter H (2004). Extended lymph node dissection for gastric cancer: who may benefit? Final results of the randomized Dutch gastric cancer group trial. J Clin Oncol Off J Am Soc Clin Oncol.

[16] Sasako M, Sakuramoto S, Katai H (2011). Five-year outcomes of a randomized phase III trial comparing adjuvant chemotherapy with S-1 versus surgery alone in stage II or III gastric cancer. J Clin Oncol Off J Am Soc Clin Oncol.

[17] Songun I, Putter H, Kranenbarg EM (2010). Surgical treatment of gastric cancer:15-year follow-up results of the randomized nationwide Dutch D1D2 trial. Lancet Oncol.

[18] Bang YJ, Kim YW, Yang HK, Chung HC, Park YK, Lee KH, Lee KW, Kim YH, Noh SI, Cho JY, Mok YJ, Kim YH, Ji J, Yeh TS, Button P, Sirzén F, Noh SH (2012). Adjuvant capecitabine and oxaliplatin for gastric cancer after D2 gastrectomy(CLASSIC): a phase 3 open-label, randomized controlled trial. Lancet.

[19] Sakuramoto S, Sasako M, Yamaguchi T, Kinoshita T, Fujii M, Nashimoto A, Furukawa H, Nakajima T, Ohashi Y, Imamura H, Higashino M, Yamamura Y, Kurita A, Arai K (2007). Adjuvant chemotherapy for gastric cancer with S-1, an oral fluoropyrimidine. N Engl J Med.

[20] Hallissey MT, Dunn AA, Ward LC (1994). The second british stomachcancer group trial of adjuvant radiotherapy or chemotherapy in resectable gastric cancer: five-year follow up. Lancet.

[21] Ml Z, Kang M, Li GC (2016). Postoperative chemoradiotherapy versus chemotherapy for R0 resected gastric cancer with D2 lymph node dissection: an up-to-date meta-analysis. World J Surg Onc.

[22] Batista TP, Mendona LM, Fassizoli-Fonte AL (2012). The role of perioperative radio-therapy in gastric cancer. Oncol Rev.

[23] Lee J, Limdo H, Kim S (2012). Phase III trial comparing capecitabine plus cisplatin versus capecitabine plus cisplatin with concurrent capecitabine radiotherapy in completely resected gastric cancer with D2 lymph node dissection: the ARTIST trial. J Clin Oncol Off J Am Soc Clin Oncol.

[24] Kim TH, Park SR, Ryu KW (2012). Phase 3 trial of postoperative chemotherapy alone versus chemoradiation therapy in stage III-IV gastric cancer treated with R0 gastrectomy and D2 lymph node dissection. Int J Radiat Oncol Biol Phys.

[25] Chen Q, Chen R, Dong Y (2020). Inhibitory effect of endostar combined with radiotherapy on gastric cancer animal models. World J Surg Onc.

[26] Zhu WG, Xua DF, Pu J, Zong CD, Li T, Tao GZ, Ji FZ, Zhou XL, Han JH, Wang CS, Yu CH, Yi JG, Su XL, Ding JX (2012). A randomized, controlled, multicenter study comparing intensity-modulated radiotherapy plus concurrent chemotherapy with chemotherapy alone in gastric cancer patients with D2 resection. Radiother Oncol J Eur Soc Ther Radiol Oncol.

[27] Bouvier AM, Créhange G, Azélie C, Cheynel N, Jouve JL, Bedenne L, Faivre J, Lepage C, Maingon P (2014). Adjuvant treatments for gastric cancer: from practice guidelines to clinical practice. Dig Liver Dis.

[28] Zhan YQ, Li W, Sun XW (2005). Long-term efficacy of surgical treatment of gastric cancer. Chinese J Surg.

[29] Wisbeck WM, Becher EM, Russell AH (1986). Adenocarcinoma of the stomach:autopsy observations with therapeutic implications for the radiation oncologist. Radiother Oncol.

[30] Roviello F, Marrelli D, de Manzoni G (2003). Porspective study of peritoneal recurrence after curative surgery for gastric cancer. Br J Surg.

[31] Maehara Y, Hasuda S, Koga T (2008). Postoperative outcome and Sites of recurrence in patients following curative resection of gastric cancer. Br J Surg.

[32] Kan Y, Li S, Zheng Y (2005). Extensive lymph node clearance for gastric cancer l0 years experience summary. Chinese J Gen Surg.

[33] Ichiyoshi Y, Toda T, Minamisono Y, Nagasaki S, Yakeishi Y, Sugimachi K (1990). Recurrence in early gastric cancer[ J]. Surgery.

[34] Sarka B, Karagol H, Gumus M (2004). Timing of death from tumor recurrence after curative gastrectomy for gastric cancer. Am J Clin Oncol.

[35] Hartgrink HH, van de Velde CJ (2005). Status of extended lymph node dissection: locoregional control is the only way to survive gastric cancer. J Surg Oncol.

[36] Brenner RM, Kivity S, Kundel Y, Purim O, Peled N, Idelevich E, Lavrenkov K, Kovel S, Fenig E, Sulkes A, Brenner B (2013). Ethnic variation in toxicity and outcome of adjuvant chemoradiation for gastric cancer in Israel. Anticancer Res.

